# Impact of Beef and Beef Product Intake on Cognition in Children and Young Adults: A Systematic Review

**DOI:** 10.3390/nu11081797

**Published:** 2019-08-03

**Authors:** Ruopeng An, Sharon M Nickols-Richardson, Naiman Khan, Jianxiu Liu, Ruidong Liu, Caitlin Clarke

**Affiliations:** 1 Brown School, Washington University, St. Louis, MO 63130, USA; 2 Department of Kinesiology and Community Health, University of Illinois, Champaign, IL 61820 USA; 3 Department of Food Science and Human Nutrition, Division of Nutritional Sciences, University of Illinois Extension, University of Illinois, Champaign, IL 61801, USA; 4 Department of Physical Education, Tsinghua University, Beijing 100084, China

**Keywords:** beef, cognition, child, young adult, review

## Abstract

(1) Background: Undernutrition and micronutrient deficiency have been consistently linked to cognitive impairment among children and young adults. As a primary source of dietary animal protein, beef consumption holds the potential to improve diet quality and positively influence cognitive function. This study systematically reviewed evidence linking beef intake to cognition among children and young adults. (2) Methods: A literature search was conducted in seven electronic bibliographic databases for studies assessing the impact of beef consumption on cognition. (3) Results: We identified eight studies reporting results from five unique interventions. Two interventions were conducted in Kenya, two in the U.S. and one in four countries including Guatemala, Pakistan, Democratic Republic of the Congo and Zambia. Only one intervention employed a non-feeding control arm and found beef consumption to improve cognitive abilities compared to the control. However, the other interventions comparing beef consumption to other food types found no consistent result. (4) Conclusions: Evidence pertaining to the impact of beef consumption on cognition remains limited due to the small and heterogeneous set of studies. Future research should adopt a population representative sample and longer follow-up period, employ a non-feeding control arm and comprehensively measure nutrient intakes among study participants.

## 1. Introduction

Undernutrition, characterized by inadequate food and nutrient intakes necessary for human growth and health, is a leading public health concern in many developing countries as well as among socioeconomically disadvantaged populations in developed nations [1,2]. Cognition is one’s ability to process information through perception and experience in an effort to learn knowledge and make decisions [3]. Undernutrition has been consistently associated with cognitive underdevelopment and impairment among children and young adults [4,5]. Iron deficiency, one of the most prevalent micronutrient deficiencies worldwide, is found to be associated with impaired cognition through its adverse interference with the brain structure [6]. Animal trials have demonstrated that brain iron is sensitive to dietary iron [7]. Similarly, human-based studies have documented a positive relationship between iron treatment and improved concentration and intelligence quotient [8]. Studies in Chile, India, Mexico and Zanzibar found iron-deficiency anemia in infants and children to be associated with poorer cognitive performance [9]. In addition, vitamin B_12_ deficiency results in neurologic deficits and impaired cognition [10], which in turn negatively impacts academic performance [11,12]. Protein deficiency may impair mental development and cognition among children, causing problems with attention, perception, motivation, motor control and responsiveness [13]. Overall, deficiencies of energy, protein and certain micronutrients (e.g., zinc, vitamin B_12_, iron and iodine) may lead to irreversible effects on neurocognitive development in children [10,11,12,14,15,16,17,18]. Moreover, undernourished children are more likely to sustain poor cognitive function as they mature, negatively impacting future educational attainment, income, physical and mental health and quality of life [19].

Adequate nutrient intake in early life is crucial for cognitive development. However, traditional diet primarily based on staple foods such as rice, wheat, corn, sorghum, roots and tubers in some low- and middle-income countries are often low in energy, protein and other micronutrients that are key for cognitive functioning [20]. In addition, these staple foods are often high in phytate and fiber, which reduces the bioavailability of certain micronutrients such as iron, calcium and zinc [21,22]. Dietary animal protein has high biological value and is rich in iron, zinc, multiple B vitamins and other essential nutrients [23]. 

As a primary source of dietary animal protein, beef consumption, especially fresh and lean beef consumption, holds the potential to improve diet quality and positively influence cognitive function in children and young adults [24]. Despite the high prevalence of global undernutrition and possible pathways linking beef consumption to cognition, no relevant review has been conducted. This study is the first that systematically reviews the scientific evidence regarding the impact of beef consumption on cognition among children and young adults. We hypothesized that provision of beef products to children and young adults prone to undernutrition would be beneficial to their cognitive functioning and performance. The study findings may shed light on policy interventions that aim to improve nutritional status and prevent undernutrition among socioeconomically disadvantaged younger populations.

## 2. Methods 

A systematic review was conducted in accordance with the Preferred Reporting Items for Systematic Reviews and Meta-Analyses [25].

### 2.1. Study Selection Criteria

Studies that met all of the following criteria were included in the review: (1) Study design: randomized controlled trials (RCT); (2) Study subjects: children and young adults aged 21 years and younger; (3) Exposure: beef and/or beef product consumption; (4) Outcomes: cognitive function and development; (5) Article type: peer-reviewed publications; (6) Time window of search: from the inception of an electronic bibliographic database to February 9, 2019; and (7) Language: articles written in English.

Studies that met any of the following criteria were excluded from the review: (1) Studies that incorporated no outcome pertaining to beef consumption in relation to cognition; (2) Studies that examined the influence of overall red meat consumption or certain dietary patterns (e.g., Mediterranean diet) on cognition without differentiating the independent effect of beef consumption; (3) Non-experimental study designs; (4) Letters, editorials, study protocols, conference proceedings, books or review articles; and (5) Articles not written in English.

### 2.2. Search Strategy

A keyword search was performed in seven electronic bibliographic databases: Academic Search Complete, Cochrane Library, PubMed, Scopus, Web of Science, Cumulative Index of Nursing and Allied Health (CINAHL) and Google Scholar. The search algorithm included all possible combinations of keywords from the following two groups: (1) “beef”; and (2) “cognition,” “cognitive,” “executive,” “dementia,” “memory,” “neurocognitive” and “neurocognition.” The MeSH terms “cognition,” “cognitive dysfunction,” “executive function,” “dementia,” “memory” and “neurocognitive disorders” were included in the PubMed search. All keywords in PubMed were searched with the “[All fields]” tag, which are processed using Automatic Term Mapping [26]. The search function TS = Topic was used in Web of Science, which launches a search for topic terms in the fields of title, abstract, keywords and Keywords Plus^®^. The search algorithm in PubMed is provided in Appendix A. Titles and abstracts of the articles identified through the keyword search were screened against the study selection criteria. Potentially relevant articles were retrieved for an evaluation of the full text. Two co-authors independently conducted the title and abstract screening and identified potentially relevant articles for the full-text review. Inter-rater agreement was assessed using the Cohen’s kappa (*κ* = 0.91). Discrepancies were resolved through face-to-face discussions between the two co-authors. Articles identified from the title and abstract screening were reviewed in full texts. The two co-authors jointly determined the final pool of articles included in the review.

A reference list search (i.e., backward reference search) and cited reference search (i.e., forward reference search) were conducted based on the full-text articles that met the study selection criteria that were identified from the keyword search. Articles identified from the backward and forward reference search were further screened and evaluated by using the same study selection criteria. Reference searches were repeated on all newly identified articles until no additional relevant articles were found.

### 2.3. Data Extraction and Synthesis

A standardized data extraction form was used to collect methodological and outcome variables from each selected study, including authors, publication year, country, sample size, age at baseline, feeding frequency, intervention duration, follow-up duration, number of repeated measures, statistical models, attrition rate, intervention arms and control, feeding methods, cognitive domains and measures and key findings. A few studies reported outcomes from the same sample [27,28,29,30]. No two studies using different samples provided quantitative estimates for the impact of beef consumption on cognition focusing on the same domain and measure of cognition. Therefore, a meta-analysis proved infeasible. We summarized the common themes and findings of the included studies narratively.

### 2.4. Study Quality Assessment

We used the National Institutes of Health’s Quality Assessment Tool for Controlled Intervention Studies to assess the quality of each included study [31]. This assessment tool rates each study based on 14 criteria. For each criterion, a score of one was assigned if ‘yes’ was the response, whereas a score of zero was assigned otherwise. A study-specific global score ranging from zero to 14 was calculated by summing up scores across all criteria. The study quality assessment helped measure the strength of scientific evidence but was not used to determine the inclusion of studies.

## 3. Results

### 3.1. Study Selection

**Figure 1:**
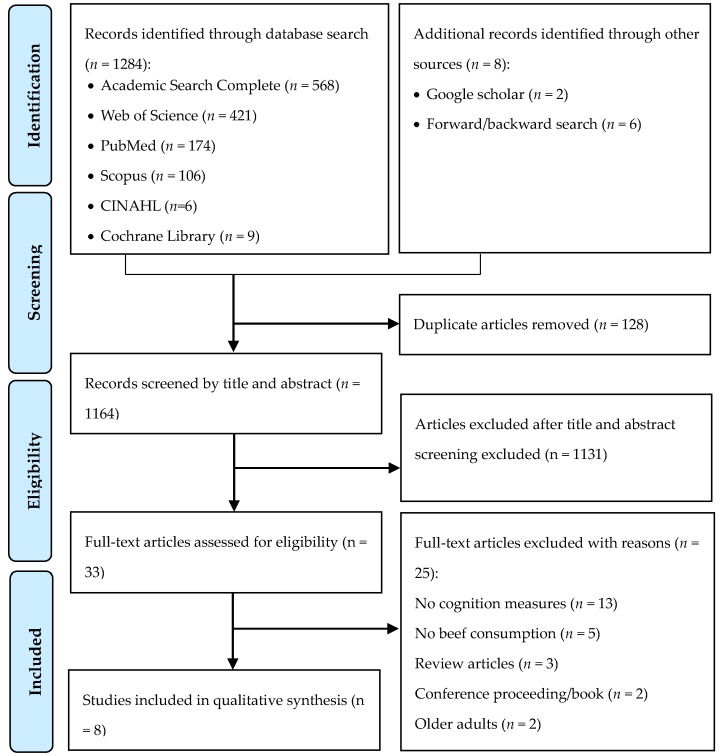
Study selection flowchart.

Figure 1 shows the study selection flow chart. We identified a total of 1292 articles through the keyword and reference search, including 568 articles from Academic Search Complete, 421 articles from Web of Science, 174 articles from PubMed, 106 articles from Scopus, nine articles from Cochrane Library, six articles from CINAHL, two articles from a hand search in Google Scholar and six articles from forward and backward search. After removing duplicates, 1164 unique articles underwent title and abstract screening, in which 1131 articles were excluded. Full texts of the remaining 33 articles were reviewed against the study selection criteria. Of these, 25 articles were excluded. The reasons for exclusion included: 13 articles did not measure cognition [32,33,34,35,36,37,38,39,40,41,42,43,44], five articles did not include beef consumption [45,46,47,48,49], three articles were reviews [50,51,52], two articles were a conference proceeding and a book [24,53] and two articles exclusively focused on premenopausal women or older adults [54,55]. The remaining eight articles that examined the effects of beef consumption on cognition among children and young adults were included [27,28,29,30,56,57,58,59]. Among these eight articles, four were based on the same study sample and intervention administered in Kenya, resulting in a total of five unique interventions to be included in the review.

### 3.2. Basic Characteristics of the Included Studies

Table 1 summarizes the basic characteristics of the eight articles, which reported results from five unique interventions. Two interventions were conducted in Kenya, two in the U.S. and one in four countries including Guatemala, Pakistan, Democratic Republic of the Congo and Zambia. Sample size totaled 1797 participants but varied substantially across studies, with a median of 88 participants and a range from 43 to 1062 participants. Two interventions recruited young children aged 4–14 years at baseline, two recruited infants aged 5–6 months and the remaining one recruited young adults aged 21 years at baseline. Two studies intervened (i.e., provision of supplementary foods such as beef or other protein-rich items) participants daily, one intervened every school day, one intervened five days per week and the remaining one intervened three days per week. Intervention duration varied from two months to two years. Study subjects were followed from 4 months to less than 3 years, with a median follow-up duration of 1.8 years. During the intervention period, a participant’s cognitive function was measured 3.5 times on average. A variety of statistical models were applied across studies, including linear regression, hierarchical regression, panel data model and mixed-effects model. Attrition rates among all studies were below 25%, with a median of 11.4% and a range from 3.5% to 23%.

Table 2 summarizes intervention arms, feeding methods and cognitive measures. One intervention implemented four arms [27,28,29,30], including the control arm without supplemental food, the vegetable snack arm with githeri plus oil-added corn, beans and vegetables, the milk snack arm with githeri plus 200 mL of milk and the beef snack arm with githeri plus 60 g of beef. The vegetable, milk and beef snacks were equicaloric (240 kcal per day). Following a year of intervention, the energy content was increased to 315 kcal per day (about 230 g of vegetable, 250 mL of milk and 85 g of beef). Krebs (2006, 2012) adopted a beef arm and a cereal arm [56,57]. In the beef arm, infants were fed with lyophilized beef product (30 g per day for infants aged 6–11 months and 45 g per day for infants aged 12–18 months); and in the cereal arm, infants were fed with a micronutrient-fortified rice cereal (70 and 105 kcal per day for the first and second 6-month periods, respectively). An infant reluctant to accept cereal had the option of mixing the food with selected fruit puree. These two feeding arms were equicaloric. Blanton (2014) focused on young women fed with beef or non-beef lunch [58]. Beef lunch consisted of 85 g of beef and non-beef lunch consisted of 85 g non-beef entrée (e.g., egg, chicken or turkey breast and cheese); whereas both included 56 g of starch and 237 mL of bottled water. Loo (2017) implemented three intervention arms of biscuits made of beef, soy or wheat. Beef biscuits added dried beef powder to the basic recipe of wheat flour [59]. Soy biscuits were made of wheat and soy flour mix. Wheat biscuits were made of wheat flour. The three types of biscuits are equicaloric, all made with wheat flour plus 4 g of protein per 100 kcal.

A total of 13 measures were applied to different cognitive domains among the selected studies. Five examined children’s cognitive performances: fluid intelligence measured by the Raven’s progressive matrices (RPM), vocabulary capacity measured by the verbal meaning test (VMT), basic arithmetic knowledge measured by the arithmetic skills test (AST), concentration, attention and immediate memory measured by the digit span test (DST), cognitive style measured by the embedded figure test (EFT), integrate visual and motor abilities measured by the Beery test of visual-motor integration (VMI) and academic performance measured by the zonal-wide multi-tests. Two studies examined infant mental, motor and behavioral development using the Bayley scales of infant development (BSID-II). One study examined young adults’ motor skill, immediate and delayed memory, spatial planning ability, working memory and sustained attention using the motor screening test (MOT), verbal recognition memory (VRM), one touch stockings of Cambridge (OTS), spatial working memory (SWM) and rapid visual information processing (RVP).

Table 3 summarizes effect estimates and main findings on beef consumption and cognition of the included studies. Intervention effectiveness can be classified into two categories—comparison between beef and the control arm and comparison between beef and the other intervention arms. All three studies found beef consumption to result in improved cognitive performance compared to the control arm. Specifically, Whaley (2003) found the beef snack arm showed greater gains on fluid intelligence (effect size (ES) = 0.34, standard error (SE) = 0.2, *p* < 0.05) and basic arithmetic knowledge (ES = 0.18, SE = 0.1, *p* < 0.05) compared to the control arm. Neumann (2007) reported the same findings on fluid intelligence and arithmetic test. In addition, the beef snack arm had higher scores in the zone-wide school final exam compared to the control arm. Hulett (2014) found the beef snack arm had higher scores in math (ES = 5.41, SE = 2.66, *p* < 0.05), English (ES = 14.3, SE = 3.34, *p* < 0.05), Kiembu (ES = 7.71, SE = 3.24, *p* < 0.05), Kiswahili (ES = 8.29, SE = 3.63, *p* < 0.05), geography (ES = 9.31, SE = 2.37, *p* < 0.05) , arts (ES = 5.26, SE = 1.82, *p* < 0.05) and total scores (ES = 57.5, SE = 16.3, *p* < 0.05).

Seven studies compared intervention effectiveness of beef consumption to other intervention arms (e.g., milk, cereal and soy biscuits). Whaley (2003) found the beef snack arm showed greater gains on fluid intelligence than the vegetable (ES = 0.41, SE = 0.2, *p* < 0.05) and milk snack arms (ES = 0.68, SE = 0.2, *p* < 0.01). In contrast, no difference was revealed in vocabulary (vegetable arm: ES = −0.09, SE = 0.22; milk arm: ES = 0.14, SE = 0.22) and basic arithmetic knowledge (vegetable arm: ES = −0.08, SE = 0.09; milk arm ES = 0.15, SE = 0.09) between the beef arm and the vegetable or milk snack arm. Neumann (2007) carried the intervention for an additional year and found the beef snack arm outperformed the milk and vegetable snack arms on basic arithmetic knowledge. Moreover, the beef snack arm performed better in fluid intelligence, zone-wide school final exams and arithmetic subtest compared to the vegetable and milk arms, whereas the vocabulary, concentration, attention and immediate memory showed no difference. Compared to the milk snack group, Hulett (2014) found that the beef snack arm outperformed on English testing (ES = 6.58, SE = 2.87, *p* < 0.05) and in comparison to the vegetable snack arm, the beef snack arm had higher testing scores in math (ES = 6.18, SE = 2.28, *p* < 0.05), English (ES = 12.5, SE = 3.14, *p* < 0.05), Kiembu (ES = 6.03, SE = 3.05, *p* < 0.05), Kiswahili (ES = 7.11, SE = 3.41, *p* < 0.05), geography (ES = 7.00, SE = 2.24, *p* < 0.05) , arts (ES = 4.67, SE = 1.71, *p* < 0.05) and total scores (ES = 44.8 SE = 12.55, *p* < 0.05). Blanton (2014) found that young adults on the beef lunch arm had improved delayed memory (*p* < 0.05), spatial planning ability and working memory (*p* < 0.05) and sustained attention (*p* < 0.05) compared to the non-beef lunch arm. Two studies that focused on infants found no difference in motor, mental and behavioral subscores in the BSID-II between the beef and cereal arms. In contrast, Loo (2017) found that HIV-affected school-age children provided with soy biscuits showed greater improvement in fluid intelligence compared to the beef biscuits arm, whereas the other cognitive measures showed no difference between the beef arm and the other arms, including DS-forward (ES = 0.13, 95% CI = −0.27, 0.52), DS-backward (ES = 0.28, 95% CI = −0.09, 0.64), DS-total (ES = 0.33, 95% CI = −0.27, 0.94), VMT (ES = 1.10, 95% CI = −0.48, 2.68), AST (ES = 0.57, 95% CI = −0.01, 1.13), EFT (ES = 0.07, 95% CI = −0.51, 0.64) and VMI (ES = 0.15, 95% CI = −0.63, 0.93). In addition, Loo (2017) found the beef biscuits arm showed no difference compared to the wheat biscuits arm in the test scores of vocabulary (ES = −0.50, 95% CI = −2.19, 1.19), concentration, attention and immediate memory (ES = 0.18, 95% CI = −0.46, 0.83), fluid intelligence (ES = −0.14, 95% CI = −1.56, 1.28), basic arithmetic knowledge (ES = 0.02, 95% CI = −059, 0.63), cognitive style (ES = −0.21, 95% CI = −0.83, 0.40) and integrate visual and motor abilities (ES = −0.52, 95% CI = −0.36, 4.48)

### 3.3. Study Quality Assessment

Table 4 reports criterion-specific and global ratings from the study quality assessment. The included studies scored 9.5 out of 14 on average, with a range from eight to 13. All studies stated that the control and intervention arms were similar at baseline on key characteristics that could affect outcomes, strictly adhered to the pre-specified intervention protocols and had outcomes assessed using valid and reliable measures. Four studies had successful randomization and the investigators assessing the outcomes were blinded to the participants’ group assignments [56,57,58,59]. Six of the studies had the overall drop-out rate lower 20% [27,28,29,30,56,57]. In contrast, none of the studies used an intention-to-treat analysis. Only two studies reported the sample size was sufficiently large to detect a difference in the main outcome between groups with an 80% statistical power [56,57].

## 4. Discussion

This study systematically reviewed scientific evidence regarding the impact of beef consumption on cognition among children and young adults. A total of five interventions were identified. All interventions compared beef or beef product intake to alterative foods such as milk snack, cereal, wheat and chicken. One intervention also included a non-feeding control arm. Domains and indicators of cognitive function assessed included fluid intelligence, vocabulary capacity, concentration, attention and immediate memory, spatial planning ability, working memory and sustained attention, basic arithmetic knowledge and academic performance. The intervention comparing beef intake to the non-feeding control arm found that beef consumption improved cognitive performance on fluid intelligence, basic arithmetic knowledge and six of the seven zone-wide school final exams. In contrast, the interventions comparing beef intake to alternative foods revealed no consistent difference. Beef consumption was found to be more effective in improving certain cognitive domains compared to the milk and the vegetable arm. On the other hand, no consistent difference was found between the beef and chicken lunch arms or between the beef and iron-fortified cereal arms. In addition, soy biscuit was found to outperform beef biscuit on fluid intelligence improvement among HIV-affected school-age children.

Possible mechanisms for changes in cognitive abilities and academic tests may contribute to the presence of micronutrients that affect learning and brain function. Through its intrinsic micronutrient content and high-quality protein, beef consumption may facilitate certain cognitive abilities such as information processing, which is essential in learning tasks and problem-solving [28]. Beef intake can increase iron and zinc absorption from fiber and phytate-rich plant staples [60]. Gewa (2009) found that the improvement in fluid intelligence was predicted by daily iron intake in children [29]. Moreover, zinc intake was associated with the improvement in concentration, attention and immediate memory over time [29]. Children with higher intake of B vitamins (e.g., B_2_ and B_12_) also experienced improved performance in concentration, attention and immediate memory compared to compared to those with lower intakes. B vitamins may affect cognitive function and development through their roles in neurotransmitter synthesis and modulation, axon and myelin sheath integrity and homocysteine metabolism [61,62,63]. Cognitive impairment in areas such as memory, reasoning and attention have been reported among vitamin B_12_ deficient children [17].

The interventions comparing beef intake to alternative foods revealed no consistent difference in their impact on cognition among children and young adults. Brain activity measured by electroencephalography (EEG) was found to be linked to iron intake and a positive relationship between iron intake and planning speed, attention and memory was documented among adolescents and young adults [64,65]. Treating iron-deficiency anemia with iron supplementation may reverse EEG abnormalities [66] and improve cognitive performance [67]. Beef is shown to outperform poultry and fish in improving serum ferritin in intervention studies involving adolescents [68] and iron-deficient women [69]. Compared to the vegetable and milk snack arm, beef snack had more nutrient content of protein, available iron, zinc and vitamin B_12_. Due to the higher nutrient intake in the beef snack arm than the milk and the vegetable arms, children in the beef snack arm improved greater in arithmetic, English, Kiembu, Kiswahili, geography and arts than the other snack arms [30]. According to Krebs (2006, 2014), no difference in the impact on infants’ cognitive development was found between the beef and iron-fortified cereal arms. The two arms had the same status of zinc and vitamin B_12_. Both anemia and iron-deficiency rates for the two arms after intervention were notably lower than those in the general population, indicating that consumption of beef as a supplementary food provides sufficient iron for infants’ growth [56]. Gewa et al. (2009) assessed the relationship between specific dietary micronutrients and gains in cognitive test scores among primary school children in rural Kenya. After controlling for confounders such as energy intake, school, socio-economic status and morbidity, iron, zinc, vitamin B_12_ and riboflavin were found to be associated with improved cognitive test scores [29]. These findings suggested that micronutrients from beef, independent of energy intake, could be an important predictor of cognitive development in children. One study focusing on HIV-affected school-age children found the soy biscuits outperformed the beef biscuits in gains related to fluid intelligence. Soy is a rich source of protein, including all essential amino acids. Children assigned to the soy biscuit intervention group received greater amounts of absorbable iron due to the high concentration of iron in soy flour [59]. Moreover, soybean is rich in flavonoids and has the potential to enhance memory and cognitive performance through their ability to protect vulnerable neurons, enhance existing neuronal function and stimulate neurogenesis [70,71]. Given the high environmental footprint of beef production, soy could serve as an alternative to beef in an effort to facilitate cognitive development in developing countries. Soy isoflavones are implicated in immune functioning [72]. It may also adjunctively preserve the neuronal functioning of HIV-1-infected persons by diminishing apoptotic signaling induced by the HIV-1 viral protein Tat [73]. Thus, it is possible that soy nutrients may have a role in improving pregnant women’s immune functioning and these effects are carried over to their children. Therefore, research conducted on healthy children may be warranted to eliminate the potential confounding effect in the comparison of effectiveness on cognitive functioning between beef and soybean intake. In addition, the intervention fed study participants with beef biscuits made of dried beef powder, which might not have the same biological properties compared to whole or fresh beef. This might have compromised the intervention effectiveness on cognitive performance.

To our knowledge, this review serves as the first attempt to synthesize scientific literature regarding the impact of beef consumption on cognition among children and young adults. However, several limitations pertaining to the review and selected studies should be noted. The randomization of the intervention, reported in the studies of Whaley (2003), Neumann (2007), Gewa (2009) and Hulett (2014), was not successful due to logistical difficulties. Four of the five interventions did not adopt a control arm, which prevented us from evaluating the effectiveness of beef intake relative to non-feeding status. The benefit of beef consumption over other foods on cognitive performance might require larger intakes over a longer duration.

The limitations pertaining to this review and the selected studies warrant future research. A small and heterogeneous set of studies were included in the review. No two studies shared the same quantitative estimate on the relationship between beef consumption and a specific cognitive domain, which made meta-analysis infeasible. Studies were conducted in different countries with diverse convenience samples of different age groups, which confined the generalizability of review findings. Beef was provided in different forms such as snacks, biscuits and lunches and in different quantities, which may exert differential impact on cognitive outcomes, a formal test of which is beyond the scope of this review. Future research should adopt a population representative sample and longer follow-up period, employ a non-feeding control arm and comprehensively measure the nutrient intakes among participants in an effort to advance research in this field.

## 5. Conclusions

In conclusion, this study systematically reviewed the relationship between beef and beef product consumption and cognition among children and young adults. Only one intervention employed a non-feeding control arm and found beef consumption to improve cognitive abilities compared to the control. However, the other interventions comparing beef consumption to other food types found no consistent result. Children supplemented with beef improved in certain cognitive domains than the milk and vegetable snack arms. No consistent effects were reported when compared beef with chicken lunch arms. No difference was reported between the beef and the iron-fortified cereal arm in two interventions. Moreover, soy biscuit was found to outperform beef biscuit on fluid intelligence improvement among HIV-affected school-age children. The overall scientific evidence remains limited due to the small and heterogeneous set of studies included in the review. Future research should adopt a population representative sample and longer follow-up period, employ a non-feeding control arm and comprehensively measure nutrient intakes among study participants.

## Figures and Tables

**Table 1:** Basic characteristics of the studies included in the review.

Study ID	First Author (year)	Country	Sample Size ^1^	Age at Baseline	Feeding Frequency	Intervention Duration (month)	Follow-Up (year)	No. of Repeated Measures	Statistical Model	Attrition Rate
1	Whaley (2003) [27]	Kenya	555 a	6–14 years	Every school day	21	2.5	4	Linear hierarchical regression	8.6%
2	Krebs (2006) [56]	USA	88 b	5 months	Monthly supply of complementary food	2	0.8	3	Hierarchical regression	18.0%
3	Neumann (2007) [28]	Kenya	Cohort I: 525 Cohort II: 375a	6–14 years	Every school day	21	2.3	4	Linear hierarchical regression	8.6%
4	Gewa (2009) [29]	Kenya	554 a	7 years	Every school day	24	2.0	4	Panel data model	4.5%
5	Krebs (2012) [57]	Guatemala, Pakistan, DR Congo, Zambia	1062 c	6 months	Daily	12	1.0	2	Linear regression	14.1%
6	Blanton (2014) [58]	USA	43 d	21 years	Three times a week	4	0.3	2	Mixed effects regression	23.0%
7	Hulett (2014) [30]	Kenya	360 e	7 years	Every school day	18	1.6	5	Panel data model	3.5%
8	Loo (2017) [59]	Kenya	49 f	4-8 years	Five days a week	18	2.0	4	Mixed effects regression	22.4%

^1^ Sample sizes superscripted with the same alphabet come from the same analytical sample.

**Table 2:** Intervention arms, feeding methods and cognition measures.

	Intervention Arms	Type/Quantity of Feeding	Feeding Specifics	Cognitive Domains	Cognitive Measures
1	4: control, vegetable snack, milk snack and beef snack	Control: no food; Vegetable snack: githeri plus corn, beans and vegetables; Milk snack: githeri plus 200 mL milk; Beef snack: githeri with 60g beef.	The snacks were equicaloric (~240 kcal/day). After 1 year, the energy content increased to ~315 kcal/d —vegetable snack to ~230 g, milk snack to 250 mL and beef snack to 85 g.	1. Perceptual, reason and comparisons (fluid intelligence); 2. Vocabulary; 3. Basic knowledge of arithmetic.	1.Raven’s Progressive Matrices (RPM); 2. Verbal Meaning test (VMT); 3. Arithmetic skills test (AST).
2	2: beef, iron-fortified cereal	Beef: beef and beef gravy with 25 mg Zn/g and 15 mg Fe/g; Cereal: iron-fortified infant rice cereal with 15 mg Zn/g and 740 mg Fe/g.	Any infant who was found to be reluctant to accept cereal feeding had the option of mixing the food with selected pureed fruits.	Mental, motor and behavior.	Bayley Scales of Infant Development (BSID-II).
3	4: control, vegetable snack, milk snack and beef snack	Control: no food; Vegetable snack: githeri plus corn, beans and vegetables; Milk snack: githeri plus 200 mL milk; Beef snack: githeri with 60 g beef.	The snacks were equicaloric (~240 kcal/day). After 1 year, the energy content increased to ~315 kcal/d —vegetable snack to ~230 g, milk snack to 250 mL and beef snack to 85 g.	1. Perceptual, reason and comparisons (fluid intelligence); 2. Vocabulary; 3. Basic knowledge of arithmetic; 4. Concentration, attention and immediate memory; 5. Academic performance.	1. Raven’ s Progressive Matrices (RPM); 2. Verbal Meaning Test (VMT); 3. Arithmetic skills test (AST); 4. Digit Span test (DST); 5. Zonal-wide multi-test (ZMT).
4	4: control, vegetable snack, milk snack and beef snack	Control: no food; Vegetable snack: githeri plus corn, beans and vegetables; Milk snack: githeri plus 200 mL milk; Beef snack: githeri with 60 g beef.	The snacks were equicaloric (~240 kcal/day). After 1 year, the energy content increased to ~315 kcal/d —vegetable snack to ~230 g, milk snack to 250 mL and beef snack to 85 g.	1. Perceptual, reason and comparisons (fluid intelligence); 2. Vocabulary; 3. Basic knowledge of arithmetic; 4. Concentration, attentionand immediate memory.	1. Raven’ s Progressive Matrices (RPM); 2. Verbal Meaning Test (VMT); 3. Arithmetic skill Test (AST); 4. Digit Span test (DST).
5	2: beef, micronutrient-fortified cereal	Beef: lyophilized beef provides 30 g/day from 6-11 m of age and 45 g/day from 12–18 month of age; Cereal: a micronutrient-fortified rice-soy, provides 70 and 105 kcal/day for the first and second 6- month periods.	Two feeding arms were equicaloric.	Psychomotor developmental and mental developmental.	The Bayley Scales of Infant Development (BSID-II).
6	2: beef and non-beef lunch	Beef lunch: consisted of 3 oz/85 g beef, 2 oz (56 g) starch and 8 oz (237 mL) bottled water; Non-beef lunch: 3 oz/85 g non-beef entrée, 2 oz (56 g) starch and 8 oz (237 mL) bottled water.	Lunches followed a 4-week cycle menu. within each lunch day, the starch food was the same for all women and the beef or non-beef entrée was the same within each lunch arm.	1. Motor skill; 2. Immediate and delayed memory; 3. Spatial planning ability and working memory; 4. Retain spatial information, working memory and devise strategy for searching task; 5. Sustained attention with a minor working memory component.	1. Motor Screening Test (MOT); 2. Verbal Recognition Memory (VRM); 3. One Touch Stockings of Cambridge (OTS); 4 Spatial Working Memory (SWM); 5. Rapid Visual Information Processing (RVP).
7	4: control, vegetable snack, milk snack and beef snack	Control: no food; Vegetable snack: githeri plus corn, beans and vegetables, ~230 g; Milk snack: githeri plus 250 mL milk; Beef snack: githeri with 85 g beef.	The snacks were equicaloric (~315 kcal/day).	Academic performance.	School end-term test: Arithmetic, English, Kiembu, Kiswahili, Science, Geography, Arts.
8	3: wheat biscuits, beef biscuits and soy biscuits	Wheat biscuits: wheat flour biscuits used as the control arm; Beef biscuits: dried beef powder was added to the basic recipe made of wheat flour; Soy biscuits: soy flour was added to the basic recipe made of wheat flour.	Isocaloric biscuits were made with wheat flour, 4.0 g protein per 100 kcal.	1. Concentration, attention and immediate memory; 2. Perceptual, reason and comparisons (fluid intelligence); 3. Vocabulary; 4. Basic knowledge of arithmetic; 5. Cognitive style; 6. Integrate visual and motor abilities.	1. Digit Span Test (DS); 2. Raven’s Progressive Matrices (RPM); 3. Verbal Meaning Test (VMT); 4. Arithmetic skill test (AST); 5. Embedded figure test (EFT); 6. Beery test of visual–motor integration (VMI).

**Table 3:** Effect estimates and main findings on beef consumption and cognition.

Study ID	Results		Main Findings	
	Intervention Effectiveness of Beef Consumption (vs. Control)	Intervention Effectiveness of Beef Consumption (vs. other Intervention Arms)	Intervention Effectiveness of Beef Consumption (vs. Control)	Intervention Effectiveness of Beef Consumption (vs. Other Intervention Arms)
1	1. Beef snack arm showed greater gains on RPM than control: ES = 0.34, SE = 0.2, *p* = 0.045; 2. Beef snack arm showed no significant difference on VMT than control: ES = 0.2, SE = 0.23; 3. Beef snack arm showed greater gains on AST than control: ES = 0.18, SE = 0.1, *p* = 0.033.	1. Beef snack arm showed greater gains on RPM than vegetable (ES = 0.41, SE = 0.2, *p* = 0.02) and milk snack arms (ES = 0.68, SE = 0.2, *p* < 0.01); 2. Beef snack arm showed no significant difference on VMT compared with vegetable (ES = −0.09, SE = 0.22) and milk snack arms (ES = 0.14, SE = 0.22); 3. Beef snack arm showed no significant difference on AST compared with vegetable (ES = −0.08, SE = 0.09) and milk snack arms (ES = 0.15, SE = 0.09).	Children fed with beef snacks showed greater gains on RPM and AST compared with control arm but no significant difference on VMT.	Children fed with beef snacks showed greater gains on RPM than vegetable and milk arms but no significant difference on VMT and AST.
2		1. The mental percentile sub-scores in the BSID-II for beef and cereal arms showed no significant difference (SMD = −0.11; 95% CI = −0.53, 0.31); 2. The motor percentile sub-scores in the BSID-II for beef and cereal arms showed no significant difference (SMD = 0.28; 95% CI = −0.14, 0.70); 3. The behavior percentile sub-scores in the BSID-II showed no significant difference (SMD = 0.41; 95% CI = −0.01, 0.84).		Motor, mental and behavior sub-scores in the BSID-II did not differ between arms. Introduction of meat as an early complementary food for exclusively breastfed infants is feasible and was associated with improved zinc intake and potential benefits.
3	1. RPM: beef snack arm increased rate was steeper than control arm; 2. AST: beef snack arms performed better over time than control arm (*p* < 0.05); 3. VMT and DS: no significant differences; 4. Zone-wide school end-term: beef snack arm increased greater than control arm; 5. Arithmetic subtest: greater percentage increased in beef snack arm than control arm.	1. RPM: beef snack arm increased rate was steeper than milk and vegetable snack arms; 2. AST: beef snack arm performed better over time than milk and vegetable snack arms; 3. VMT and DS: no significant differences; 4. Zone-wide school end-term scores: beef snack arm performed better than milk and vegetable snack arms; 5. Arithmetic subtest: greater percentage increased in beef snack arm than vegetable and milk snack arms.	Beef snack arm showed steeper rate of increasing on RPM, AST, zone-wide school end-term total scores and arithmetic subtest scores than control arm.	Beef snack arm showed steeper rate of increase on RPM, AST, zone-wide school end-term total and arithmetic subtest scores than milk and vegetable snack arm.
5		1. Psychomotor developmental index: 99.1 (95% CI: 97.9, 100.3) and 99.7 (95% CI: 98.8, 100.7) (*p* = 0.54) for beef and cereal arms. 2. Mental developmental index: 95.2 (95% CI: 94.2, 96.2) and 95.3 (95% CI: 94.5, 96.2) (*p* = 0.82) for beef and cereal arms.		No significant different was found in the index of BSID-II in beef and cereal arms.
6		1. VRM: lunch arm had significant main effects on free recall of correct targets, with more words recalled by women in beef arm than non-beef arm (*p* = 0.007); 2. SWM: latency to first response was different (*p* = 0.0003), speed was greater in non-beef arm than beef arm; token search time was affected by different arms (*p* = 0.003). SWM strategy showed a significant effect of arm (*p* = 0.018) with better strategy showed in non-beef than beef arm; 3. RVP: lunch arm had no significant effect on latency to respond but more total hits were achieved in beef arm than non-beef arm (*p* = 0.0038), total misses were lower in beef arm than non-beef arm (*p* = 0.006), correct rejections were higher in beef arm than non-beef arm (*p* = 0.009).		Lunch arm had no consistent main effects on test performance. Beef arm performance better on VRM and RVP. Overall, the current findings do not show that intake of beef improves cognitive performance in women with decreased iron status to a greater degree than non-beef protein foods.
7	Beef snack arm showed difference with control arm on scores of Arithmetic (ES = 5.41, SE = 2.66, *p* < 0.05), English (ES = 14.3, SE = 3.34, *p* < 0.05), Kiembu (ES = 7.71, SE = 3.24, *p* < 0.05), Kiswahili (ES = 8.29, SE = 3.63, *p* < 0.05), Geography (ES = 9.31, SE = 2.37, *p* < 0.05) , Arts (ES = 5.26, SE = 1.82, *p* < 0.05) and total scores (ES = 57.5, SE = 16.3, *p* < 0.05).	Beef snack arm showed difference with milk snack arm on score of English (ES = 6.58, SE = 2.87, *p* < 0.05); Beef snack arm showed difference with vegetable snack arm on the score of Arithmetic (ES = 6.18, SE = 2.28, *p* < 0.05), English (ES = 12.5, SE = 3.14, *p* < 0.05), Kiembu (ES = 6.03, SE = 3.05, *p* < 0.05), Kiswahili (ES = 7.11, SE = 3.41, *p* < 0.05), Geography (ES = 7.00, SE = 2.24, *p* < 0.05) , Arts (ES = 4.67, SE = 1.71, *p* < 0.05) and total scores (ES = 44.8 SE = 12.55, *p* < 0.05).	Children fed with beef snack showed improvements in scores in six of the seven subjects (Arithmetic, English, Kiembu, Kiswahili, Geography and Arts) and overall total test scores compared with control arm.	Children fed with beef snack showed improvements in scores in English compared with milk snack arm; Children fed with beef showed improvements in scores in six of the seven subjects (Arithmetic, English, Kiembu, Kiswahili, Geography and Arts) and overall total test scores compared with vegetable snack arm.
8		Soy biscuits arm showed no significant with beef arm on seven of the tests including DS-forward (ES = 0.13, 95% CI: −0.27,0.52), DS-backward (ES = 0.28, 95% CI: −0.09,0.64), DS-total (ES = 0.33, 95% CI: −0.27,0.94), VMT (ES = 1.10, 95%CI: −0.48,2.68), AST (ES = 0.568, 95% CI: −0.01,1.13), EFT (ES = 0.07, 95% CI: −051,0.64), VMI (ES = 0.15, 95% CI: −0.63,0.93), except for RPM (ES = 1.87, 95% CI: 0.56,3.18, *p* < 0.05). Beef biscuits arm showed no difference with wheat biscuits arm on all of tests including DS-forward (ES = 0.09, 95% CI: −0.33, 0.51), DS-backward (ES = 0.14, 95% CI: −0.25, 0.54), DS-total (ES = 0.18, 95% CI: −0.46, 0.83), RPM (ES = −0.14, 95% CI: −1.56, 1.28), VMT (ES = −0.50, 95% CI: −2.19,1.19), AST (ES = 0.02, 95% CI: −0.59, 0.63), EFT (ES = −0.21, 95% CI: −0.83, 0.40), VMI (ES = −0.52, 95% CI: −0.36, 4.48).	HIV-affected school-age children provided with beef biscuits showed no significant difference on all of the cognitive tests compared with wheat biscuits arm.	HIV-affected school-age children provided with soy biscuits showed greater improvement in nonverbal cognitive (fluid intelligence) performance compared with beef biscuits arm.

RPM denotes Raven’s Progressive Matrices; VMT denotes Verbal Meaning test; AST denotes Arithmetic skills test; BSID-II denotes Bayley Scales of Infant Development; RVP denotes Rapid Visual Information Processing; DST denotes Digit Span Test; ZMT denotes Zonal-wide multi-test; MOT denotes Motor Screening Test; VRM denotes Verbal Recognition Memory; OTS denotes One Touch Stockings of Cambridge; SWM denotes Spatial Working Memory; RVP denotes Rapid Visual Information Processing; EFT denotes Embedded figure test; VMI denotes Beery test of visual-motor integration.

**Table 4:** Study Quality Assessment.

Criteria	1	2	3	4	5	6	7	8
1. Was the study described as randomized, a randomized trial, a randomized clinical trial or an RCT?	1	1	1	1	1	1	1	1
2. Was the method of randomization adequate (i.e., use of randomly generated assignment)?	0	1	0	0	1	1	0	1
3. Was the treatment allocation concealed (so that assignments could not be predicted)?	0	1	0	0	1	0	0	1
4. Were study participants and providers blinded to treatment group assignment?	0	0	0	0	1	0	0	1
5. Were the people assessing the outcomes blinded to the participants' group assignments?	0	1	0	0	1	1	0	1
6. Were the groups similar at baseline on important characteristics that could affect outcomes (e.g., demographics, risk factors, co-morbid conditions)?	1	1	1	1	1	1	1	1
7. Was the overall drop-out rate from the study at endpoint 20% or lower of the number allocated to treatment?	1	1	1	1	1	0	1	0
8. Was the differential drop-out rate (between treatment groups) at endpoint 15 percentage points or lower?	1	1	1	1	1	1	1	0
9. Was there high adherence to the intervention protocols for each treatment group?	1	1	1	1	1	1	1	1
10. Were other interventions avoided or similar in the groups (e.g., similar background treatments)?	1	1	1	1	1	1	1	1
11. Were outcomes assessed using valid and reliable measures, implemented consistently across all study participants?	1	1	1	1	1	1	1	1
12. Did the authors report that the sample size was sufficiently large to be able to detect a difference in the main outcome between groups with at least 80% power?	0	1	0	0	1	0	0	0
13. Were outcomes reported or subgroups analyzed pre-specified (i.e., identified before analyses were conducted)?	1	1	1	1	1	1	1	1
14. Were all randomized participants analyzed in the group to which they were originally assigned, that is, did they use an intention-to-treat analysis?	0	0	0	0	0	0	0	0
Total scores	8	12	8	8	13	9	8	10

1 denotes Yes and 0 denotes No.

## Appendix A

Search Algorithm in PubMed

“beef” AND (“cognition” OR “Cognition” [Mesh] OR “Cognitive Dysfunction”[Mesh] OR “cognitive” OR “executive” OR “Executive Function”[Mesh] OR “dementia” OR “Dementia”[Mesh] OR “memory” OR “Memory”[Mesh] OR “neurocognitive” OR “neurocognition” OR “Neurocognitive Disorders”[Mesh])
